# Skin cancer prevalence among outdoor activity participants from Queensland, Australia: aquatic *versus* land-based sun exposure

**DOI:** 10.7717/peerj.21278

**Published:** 2026-06-04

**Authors:** Ian J. Miller, Michael Stapelberg, Jeremy Hudson, Paul Coxon, Nathaniel Milani, Nedeljka Rosic, Joe Walsh, Mike Climstein

**Affiliations:** 1 Aquatic Based Research, Faculty of Health, Southern Cross University, Bilinga, Queensland, Australia; 2 Skin Clinic Robina, Gold Coast, Queensland, Australia; 3 North Queensland Skin Centre, Townsville, Queensland, Australia; 4 Biomedical Sciences, Faculty of Health, Southern Cross University, Bilinga, Queensland, Australia; 5 Clinical and Health Services Research Group, Faculty of Health, Southern Cross University, Bilinga, Queensland, Australia; 6 Faculty of Science and Engineering, Southern Cross University, Biling, Queensland, Australia; 7 Sport Science Institute, Sydney, New South Wales, Australia; 8 AI Consulting Group, Sydney, New South Wales, Australia; 9 Physical Activity, Lifestyle, Ageing and Wellbeing Faculty Research Group, University of Sydney, Sydney, New South Wales, Australia

**Keywords:** Exercise physiology, Keratinocyte carcinoma, Public health, Radiation protection, Skin Neoplasms

## Abstract

**Background:**

To determine the prevalence of keratinocyte cancer and melanoma among people conducting aquatic and non-aquatic outdoor activities, and their associated ultraviolet radiation (UVR) exposures.

**Methods:**

This was a cross-sectional study incorporating a survey and total body skin cancer screen. There were 1,403 participants attending a specialized skin cancer-focused general practice during the period of September 2021 to August 2024. The main outcomes measured were the point prevalence of pre-cancerous skin lesions, keratinocyte cancers (KC) (*i.e.*, basal cell carcinoma (BCC) and squamous cell carcinoma (SCC)) and primary cutaneous melanoma (MM).

**Results:**

Of 1,403 participants (739 women, 664 men), 512 were identified as aquatic and 891 were identified as non-aquatic. Among aquatic participants, 98 of 512 (19.1%) participants were diagnosed with BCC, compared with 142 of 891 (15.9%) non-aquatic participants. Regarding SCC in the population, 46 of 512 (9.0%) aquatic participants were diagnosed compared with 89 of 891 (10.0%) non-aquatic participants. For melanoma (MM), 48 out of 512 (9.4%) aquatic participants were diagnosed and 48 of 891 (5.4%) non-aquatic participants, resulting in an odds ratio nearly double (1.8 times) for aquatic participants compared with non-aquatic. Participants of increased age and male gender had an increased likelihood of being diagnosed with MM. Primary locations for KC were the head, face and neck (32% aquatic, 36% non-aquatic), which differed for MM, being primarily located on the back (40% aquatic, 37% non-aquatic).

**Conclusions:**

While the prevalence of BCC and SCC was similar across activity groups, aquatic participants demonstrated a significantly higher prevalence of MM, despite reporting lower cumulative lifetime UV exposure. A higher MM risk is likely attributable to greater participant engagement during peak ultraviolet radiation (UVR) hours. These findings underscore the need for skin cancer surveillance in aquatic enthusiasts, who should be recognised as a high-risk population within the national melanoma screening framework.

## Introduction

Elevated prevalence of both non-melanoma or keratinocyte cancers (KC) and melanoma (MM) has been previously reported in Australian outdoor recreational activities such as surfing and swimming (Climstein et al., 2022; Miller et al., 2023). Ausplay, a large-scale Australian national survey, reported that aquatic activities (surfing and swimming) and walking have approximately 4.6 and 9.4 million participants, respectively (AusPlay, 2023). Being active outdoors has a positive impact on health (Manferdelli, La Torre & Codella, 2019); however, increased exposure to ultraviolet radiation (UVR) is a recognised mechanism for the development of many skin-related cancers, such as basal cell carcinoma (BCC), squamous cell carcinoma (SCC) and MM (Narayanan, Saladi & Fox, 2010).

The number of MM identified in Australia was recently reported to be 18,964 annually, or 69.8 cases per 100,000 people, with 1,400 deaths (AIHW, 2024). Historically, skin-based cancers have been difficult to quantify (*e.g.*, variability in clinical presentation, lack of standardised reporting); however, it has been estimated that 69% of Australians will have a confirmed KC within their lifetime (Olsen et al., 2022). Combined, KC and MM cost the Australian health care system $1.7 billion every year (Olsen et al., 2022; AIHW, 2022). This cost is expected to increase significantly as the incidence of melanoma is projected to double by the year 2040 (Arnold et al., 2022).

To date, only a limited number of skin cancer screening studies have been conducted in Australia among outdoor recreational populations, and those available have been constrained by relatively small sample sizes (*n* = 171 to *n* = 423) (Climstein et al., 2022; Miller et al., 2023). Both studies reported elevated KC and MM incidence in predominantly water sport cohorts compared with the Australian general population. However, these studies were limited by sample size, activity-specific cohorts, and the absence of direct comparator groups, restricting inference regarding differential risk across outdoor activity types. The elevated prevalence of skin-related cancers in outdoor enthusiasts highlights the risk of UVR exposure in a nation well regarded as a sunburnt country. Despite extensive documentation of melanoma incidence by age and sex, there is a critical gap in comparative evidence examining whether different outdoor activity modalities confer different skin cancer risk. In particular, direct comparisons between aquatic and non-aquatic outdoor cohorts using standardized clinical screening are lacking.

While national data have documented age-related trends in melanoma incidence, particularly a decline among individuals under 30 years of age, there remains a paucity of research directly comparing skin cancer rates between aquatic and non-aquatic cohorts. As such, the aim of this study was to conduct a large-scale screening of outdoor enthusiasts, aquatic and non-aquatic, to quantify the prevalence of KC and MM within these cohorts.

## Materials & Methods

### Study design

The design was a cross-sectional observational study including a survey and whole-body skin cancer examination. This study followed reporting guidelines as outlined by the Strengthening the Reporting of Observational Studies in Epidemiology (STROBE) (Von Elm et al., 2007). This study was conducted within the Australian primary healthcare system, where skin cancer detection and management are commonly delivered through general practice with clinicians with additional training in skin cancer management.

These clinics operate within community primary care and are typically staffed by general practitioners with advanced training in dermoscopy and skin cancer diagnosis. Patients may attend *via* self-referral or referral from a general practitioner, often for routine skin surveillance or assessment of specific lesions. All clinical assessments and biopsies performed in this study formed part of standard clinical care, with histopathological analysis conducted through accredited pathology services.

### Ethics approval

Approval for this study was granted by Southern Cross University’s Human Research Ethics Committee (11 May 2020/47).

### Survey and exposure assessment

The survey followed the protocol previously described by Climstein et al. (2022) and Miller et al. (2023), with standardised items designed to capture demographic characteristics (age, sex, years of participation in outdoor activity), sun-exposure behaviours, and skin cancer risk factors. Participants reported their primary outdoor activity (defined as the activity occupying the greatest proportion of their outdoor time), which was used to classify them as aquatic (*e.g.*, surfing, swimming) or non-aquatic (*e.g.*, walking, running, cycling).

Participants were asked to estimate their average weekly hours spent in outdoor activity during the previous 12 months, which was used to derive annual outdoor exposure (hours/year). Estimated lifetime outdoor exposure was calculated by multiplying self-reported average annual exposure by the number of years of participation in outdoor activity. Exposure during peak UVR periods was assessed by asking participants whether they typically engaged in their activity between 10:00 and 15:00, corresponding to peak UV index conditions in most regions of Australia, and the approximate proportion of activity performed during this time.

Additional survey items captured sun-protective behaviours, including regular use of sunscreen or zinc-based products, use of protective clothing (*e.g.*, rash vest). Family and personal history of skin cancer were also recorded.

### Screening strategy

Participants were recruited from patients attending a skin examination at a primary care skin cancer clinic between September 2021 to August 2024. Inclusion criteria included patients over the age of 18 and those who identified with at least one hour of outdoor activities per week. Following ethics approval (2020/047), written consent was obtained prior to participating in the study. Participants were adult patients attending a specialist skin cancer clinic in southeast Queensland, Australia, a region characterized by high ambient UVR exposure year-round. Patients attending such clinics typically include individuals with fair skin types, extensive outdoor exposure, personal or family history of skin cancer and heightened concern regarding skin cancer risk.

Participants were queried on what physical, recreational or sporting activity they spent most time participating in outdoors, categorised as either aquatic (primarily water-based) or non-aquatic (predominantly land-based) activities. The participants who engaged in various outdoor aquatic activities were primarily surfers and swimmers. The non-aquatic participants consisted of walkers, runners and cyclists. When a participant indicated they were active in both aquatic and non-aquatic modalities, the activity where they experienced the majority of their outdoor exposure (greater than 50%) was assigned.

Following each participant’s completion of the survey, the clinician completed the last section of the questionnaire, which determined the participants’ Fitzpatrick skin type. The clinician who performed the skin examinations had a minimum of 10 years’ experience in skin cancer detection, consistent with Australian primary care models where trained general practitioners play a central role in KC and MM detection.

The clinician then completed a whole-body skin examination utilising high-resolution digital dermatoscopy with software inbuilt with artificial intelligence (Moleanalyzer-Pro, FotoFinder Systems GMbH, Bad Birnbach, Germany). The dermatoscope as part of the system was a Medicam 1,000 providing magnification of skin lesions up to 40 times. Suspicious skin lesions were identified, and the locations and suspected diagnosis were documented. Where indicated and following patient consent, a biopsy of the suspicious skin lesion was performed with subsequent histopathological confirmation to determine the presence or absence of malignancy (*e.g.*, BCC, SCC, MM).

### Statistical analysis

A combination of the Statistical Package for the Social Sciences (SPSS, the Ver. 28.0; IBM Corp., Armonk, NY, USA) and R (The R Foundation for Statistical Computing, version 4.4.1) was used to complete all statistical analyses and data visualisation. For continuous data, an independent sample *t*-test was used to assess the significance between the two groups and categorical variables were examined for significance with a chi-squared test. Crude odds ratios were calculated from univariable models, and adjusted odds ratios were derived from multivariable logistic regression adjusting for age and sex. Statistical significance was set *a priori* at *p* < 0.05.

## Results

**Figure 1:**
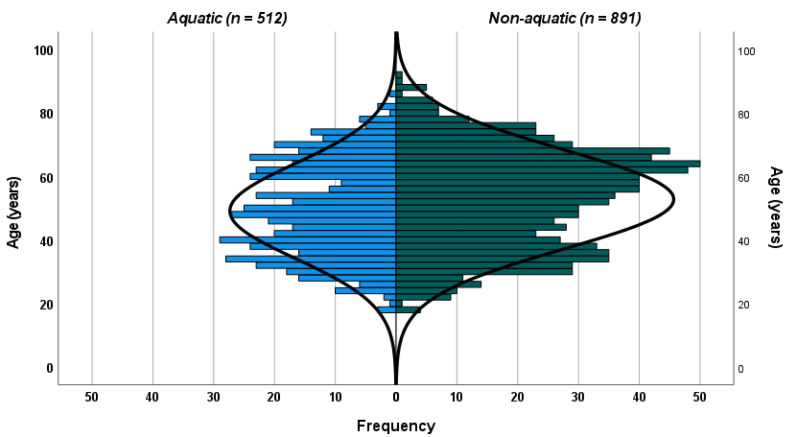
Population pyramid showing the age and sex distributions of study participants in aquatic and non-aquatic activity groups. Note: Values represent numbers of participants; the central reference line indicates parity between groups.

A total of 1,403 participants (512 aquatic, 891 non-aquatic) completed the survey and a full-body skin examination with no adverse events. Figure 1 illustrated the age and sex distribution of the study population. The population pyramid demonstrates a predominance of middle-aged and older adults, with a higher proportion of males in the aquatic group and a higher proportion of females in the non-aquatic group, consistent with the significant gender differences observed between activity groups (Fig. 1). The average age of participants was 51.4 years, with the aquatic participants younger than the non-aquatic participants (49.1 years aquatic, 52.8 years non-aquatic; *p* < .001). The distribution of gender was similar (male 47.3%, female 52.7%), with the aquatic participants being significantly more likely to be male (male 68.6%, female 31.4%; *p* < .001) and the non-aquatic participants were more likely to be female (male 35.1%, female 64.9%; *p* < .001).

On average, the aquatic participants reported fewer yearly outdoor activity exposure hours than their non-aquatic counterparts (aquatic 206.6 h, non-aquatic 254.2 h; *p* < .001), which corresponded with a significantly lower estimated lifetime exposure (aquatic 6543.7 h, non-aquatic 9565.9 h; *p* < .001). However, aquatic participants were significantly more likely to spend their outdoor activity during peak UV Index hours (aquatic 84.2%, non-aquatic 55.1%; *p* < .001) and reported a greater proportion of their total activity time during this period (35.7% aquatic, 22.6% non-aquatic; *p* < .001). Regular use of sunscreen or zinc-based photoprotection was reported by 93.0% of aquatic participants compared with 85.1% of non-aquatic participants (*p* < .001). In contrast, regular use of protective clothing was significantly lower among aquatic participants (76.8%) than non-aquatic (98.0%) participants (*p* < .001).

Approximately one-half of participants (51.1%) reported a family history of either KC or MM (aquatic 52.1%, non-aquatic 50.5%). Regarding personal history of either skin cancer or melanoma, 42.9% of participants (aquatic 43.4%, non-aquatic 42.6%) noted at least one diagnosed cancerous skin lesion prior to their skin examination (Table 1). A total of 240 participants (98 aquatic, 142 non-aquatic) were identified with at least one BCC. Of this total, 155 participants were diagnosed with a single BCC (69 aquatic, 86 non-aquatic), and 85 participants presented with multiple BCCs (29 aquatic, 56 non-aquatic). There were 135 participants (46 aquatic, 89 non-aquatic) with at least one SCC. A total of 100 participants had one SCC (35 aquatic, 65 non-aquatic), with 35 participants (11 aquatic, 24 non-aquatic) diagnosed with multiple lesions. Regarding the number of participants with MM, there were 96 participants with either one or multiple MM. There were 87 participants with one MM (45 aquatic, 42 non-aquatic), eight participants with two (two aquatic, six non-aquatic) and a single aquatic participant with three MM (Table 2).

**Table 1:** Participant demographics. Experience (years) refers to the self-reported number of years participants have engaged in their primary outdoor activity. Yearly reported exposure represents the self-reported average number of hours per year spent participating in the participant’s primary outdoor activity. Estimated lifetime exposure was calculated as years of activity multiplied by yearly reported exposure and represents an estimated cumulative exposure. Activity during peak UV indicates participation occurring between 10:00 and 15:00, corresponding to periods when the UV index typically exceeds 3 in Queensland. Amount of activity during peak UV times represents the proportion (%) of total reported outdoor activity undertaken between 10:00 and 15:00. Regular sunscreen or zinc use was defined as self-reported use during most (>50%) outdoor activity sessions. Regular clothing use refers to the routine use of sunprotective garments (*e.g.*, long sleeves, rash vests) during outdoor activity. Family history of skin cancer refers to a self-reported history of keratinocyte cancer or melanoma in firstdegree relatives. Previous history of skin cancer refers to a self-reported prior diagnosis of basal cell carcinoma, squamous cell carcinoma, or melanoma. All exposure and behaviours variables were self-reported.

Parameter	Total participants (*n* = 1,403)	Aquatic (*n* = 512)	Non-Aquatic (*n* = 891)	*P*-value
Age (years)	51.4	49.1 [47.8–50.4]	52.8 [51.8–53.8]	<.001
Gender	664 males (47.3%) 739 females (52.7%)	351 male (68.6%) 161 female (31.4%)	313 males (35.1%) 578 females (64.9%)	<.001
Experience (years) Of activity	32.6	28.8 [27.3–30.4]	34.8 [33.8–35.8]	<.001
Yearly reported exposure	236.6 h	206.6 h [180.6–226.1]	254.2 h [240.0–270.9]	<.001
Estimated lifetime exposure	8,457.3 h	6,543.7 h [5518.8–7594.4]	9,565.9 h [8798.1–10333.6]	<.001
Activity during peak UV	922 (65.7%)	431 (84.2%)	491 (55.1%)	<.001
Amount of activity during peak UV times	27.4%	35.7% [33.1–38.4]	22.6% [20.6–24.5]	<.001
Regular sunscreen or zinc use	1,234 (88.0%)	476 (93.0%)	758 (85.1%)	<.001
Regular Clothing use	1,266 (90.2%)	393 (76.8%)	873 (98.0%)	<.001
Family history of skin cancer	717 (51.1%)	267 (52.1%)	450 (50.5%)	.553
Previous history of skin cancer	602 (42.9%)	222 (43.4%)	380 (42.6%)	.796

**Table 2:** Distribution of lesion type by participant and activity group.

	Number of participants with BCC (n)			Number of participants with SCC (n)			Number of participants with MM (n)		
Number of lesions	Aquatic	Non-aquatic	Total	Aquatic	Non-aquatic	Total	Aquatic	Non-aquatic	Total
1	69	86	155	35	65	100	45	42	87
2	18	31	49	6	20	26	2	6	8
3	4	14	18	4	1	5	1	–	1
4	3	5	8	–	2	2	–	–	–
5	3	2	5	–	1	1	–	–	–
6	–	3	3	1	–	1	–	–	–
13	1	1	2	–	–	–	–	–	–
Total	98	142	240	46	89	135	48	48	96

Aquatic participants were found to be 1.8 times more likely (*p* = .004) to have a MM when compared with the non-aquatic participants (Table 3). Of the MM lesions identified, most were *in situ* (*n* = 84, 79.2%) with the remainder categorised as invasive (*n* = 22, 20.8%). The sub-types of MM lesions classified by histopathology were ‘lentigo maligna’ (*n* = 10), ‘lentiginous’ (*n* = 6) and ‘superficial spreading’ (*n* = 5). The remaining histopathology did not further specify the MM lesion subtype (*n* = 46), grouped MM as ‘not otherwise specified’ (*n* = 23) or early evolving MM *in situ* (*n* = 16).

**Table 3:** Participants with malignancies. Odds ratios derived from multivariable logistic regression adjusting for age and sex.

	Total (*n* = 1,403)	Aquatic (*n* = 512)	Non-aquatic (*n* = 891)	*P*-value	Odds ratio
Participants with actinic keratosis	440 (31.3%)	170 (33.2%)	270 (30.3%)	.260	1.143 [.906–1.443]
BCC	240 (17.1%)	98 (19.1%)	142 (15.9%)	.125	1.249 [.940–1.659]
SCC	135 (9.6%)	46 (9.0%)	89 (10.0%)	.539	.890 [.612–1.293]
Melanoma	96 (6.8%)	48 (9.4%)	48 (5.4%)	.004	1.817 [1.199–2.754]

**Figure 2:**
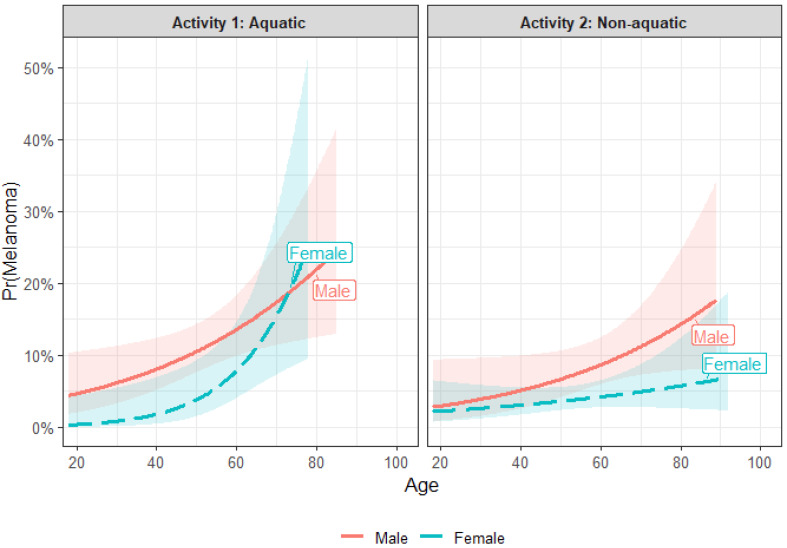
Adjusted probability of melanoma diagnosis per participant according to activity type (aquatic *vs.* non-aquatic), age, and sex. Note: Shaded regions represent 95% confidence intervals derived from multivariable logistic regression.

When adjusting for covariates including age, gender and activity type, all three variables independently influenced the likelihood of a MM being diagnosed. Figure 2 presents the adjusted probability of melanoma by activity group after accounting for age and sex. The model demonstrates that aquatic participation, increasing age, and male sex independently contribute to a higher probability of melanoma, with aquatic participants showing a consistently higher predicted risk across the age range.

**Figure 3:**
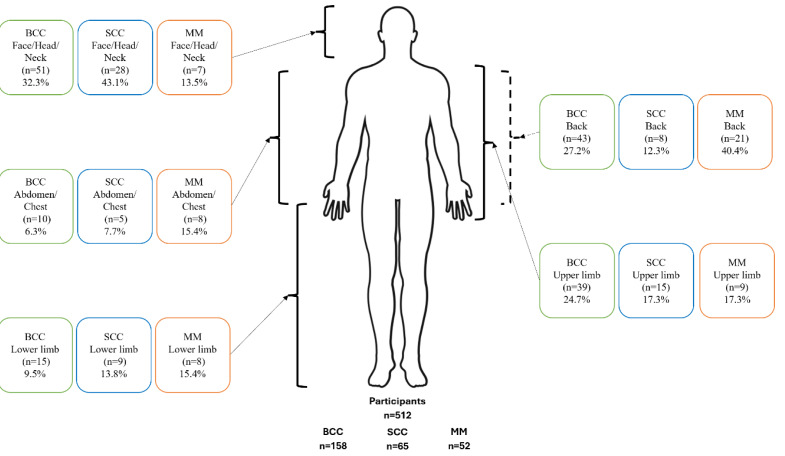
Anatomical distribution of diagnosed skin cancer lesions (basal cell carcinoma, squamous cell carcinoma, and melanoma) among aquatic participants. Percentages refer to the proportion of total lesions, not participants, BCC, basal cell carcinoma; MM, melanoma; SCC, squamous cell carcinoma.

Locations of skin cancers varied depending on the participants’ activity. For aquatic participants, BCC was most likely to be diagnosed on the face, head and neck (32.3%), followed by the back (27.2%), upper limb (24.7%) and lower limb (17.3%). Likewise, the primary locations of SCC were diagnosed on the face, head and neck (43.1%), but then upper limb (17.3%), lower limb (13.8%) and back (12.3%). The abdomen and chest had the lowest amount of diagnosed MM (6.3% BCC, 7.7% SCC). For MM, it was diagnosed overwhelmingly on the back (40.4%), followed by the upper limb (17.3%), lower limb (15.4%), abdomen and chest (15.4%), and the face, head and neck (13.5%) (Fig. 3).

**Figure 4:**
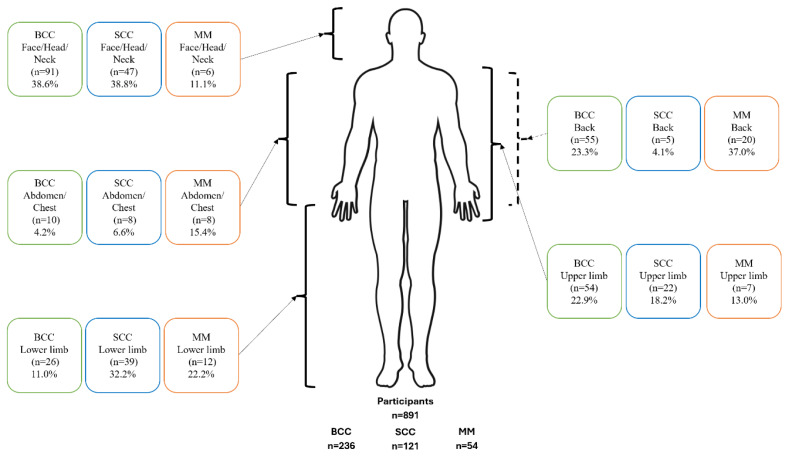
Anatomical distribution of diagnosed skin cancer lesions (basal cell carcinoma, squamous cell carcinoma, and melanoma) among non-aquatic participants. Percentages refer to the proportion of total lesions, not participants, BCC, basal cell carcinoma; MM, melanoma; SCC, squamous cell carcinoma.

For non-aquatic participants, BCC was most likely to be diagnosed on the face, head and neck (38.6%), then the back (23.3%), upper limb (22.9%), lower limb (11.0% and abdomen and chest (4.2%). For SCC, the face, head and neck (38.8%) was the primary diagnosis site, followed by the lower limbs (32.2%), upper limb (18.2%), abdomen and chest (6.6%), followed lastly by the back (4.1%). The majority of MM was diagnosed on the back (37.0%), followed by the lower limbs (22.2%), abdomen and chest (15.4%), upper limbs (13.0%) and lastly on the face, head and neck (11.1%) (Fig. 4).

## Discussion

The prevalence of skin cancer among outdoor enthusiasts has previously been reported to be significantly elevated, with MM rates many times higher (100-fold) than those observed in the general Australian population (Climstein et al., 2022; Miller et al., 2023). The present study supports previous evidence, showing that participation in outdoor recreational pursuits, particularly aquatic-based activities, was associated with a heightened risk of skin cancer. Ultraviolet radiation continues to be the predominant environmental determinant of cutaneous malignancies, including MM. Increasing age and being born male are confounding variables attributing to higher risk of developing MM over an individual’s lifetime.

The main finding of this study was the significantly elevated odds ratio for MM among aquatic participants (OR 1.8), corresponding to nearly double the risk compared with non-aquatic participants. Importantly, this association was observed despite aquatic participants reporting fewer cumulative lifetime hours of outdoor exposure. Given the reliance on self-reported exposure estimates and the cross-sectional nature of the study, this finding should be interpreted cautiously. Rather than representing a true paradox, the data suggests a potential influence of exposure timing and intensity, whereby shorter but more intense or peak period UVR exposure may contribute disproportionately to MM risk.

One plausible explanation is that MM risk may be influenced not only by cumulative UVR dose but also by the temporal pattern and intensity of sun exposure, especially during periods of peak UVR. However, this hypothesis cannot be directly tested within the present study design. Aquatic participants were more likely to engage in their activity during periods of peak solar intensity, which may partly explain the observed association with higher MM prevalence despite shorter overall exposure durations. These findings highlight the potential role of both timing and intensity of UVR exposure in melanoma pathogenesis, particularly when the UV Index exceeds 3 (Neale et al., 2024). While the consistent use of personal protective measures such as broad-spectrum sunscreen and protective clothing remains vital, behavioural modification strategies should emphasise the complementary, not substitutive role of sunscreen and clothing, particularly during prolonged or peak UVR exposure.

The anatomical distribution of MM in this cohort is also noteworthy. The primary location of MM in our study was diagnosed on the trunk (back, chest and abdomen), which is similar to the findings of the QSkin study, a randomised cohort study which included 44,000 Queensland (Australia) men and women reported on MM (Pandeya et al., 2023). When we compared our findings to the QSkin study, we noted a higher percentage of MM in the trunk for both our aquatic and non-aquatic populations (56% aquatic, 54% non-aquatic *vs.* 37.5% QSkin). While these anatomical patterns are consistent with differences in clothing coverage and activity posture, particularly for aquatic enthusiasts, these associations should be interpreted as correlative. Detailed data on site-specific exposure, clothing coverage over time, and sunscreen application by body region were not available and warrant investigation in future studies.

Both BCCs and SCCs were more likely to develop on the back of an aquatic participant (27.2% (BCC), 12.3% (SCC)) when compared to non-aquatic participants (23.3% (BCC), 4.1% (SCC)), whilst both groups were significantly higher than a large Australian population-based study (17.1% (BCC), 2.0% (SCC)) (Subramaniam et al., 2017). Whilst non-aquatic participants developed more SCCs on the lower limb than aquatic participants. This could be attributed to direct exposure of the skin on the back whilst lying prone on a surfboard, and the lower limb being submerged underwater. Actinic keratosis were common across both aquatic and non-aquatic groups, affecting one-third of participants, with no significant difference between aquatic and non-aquatic cohorts.

Sunscreen was used more often by the aquatic participants (93.0%) of our study compared to the non-aquatic participants (85.1%). However, clothing as a sun protective measure was not well reported in the aquatic participants when compared with the non-aquatic participants. Importantly, the feasibility of protective clothing differs substantially between activity types, with aquatic participants often limited by performance, comfort, and thermal considerations. This variability likely reflects a combination of behavioural, environmental and activity-specific factors, rather than misunderstanding alone. For example, aquatic environments introduce additional UVR exposure through reflection from water and surrounding surfaces (*e.g.*, sand) while prolonged water immersion may reduce sunscreen adherence and effectiveness. Together, these environmental and activity-specific factors, along with practical challenges such as maintaining adequate sunscreen coverage during prolonged or water-based activity and variability in reapplication, contribute to differences in effective photoprotection (Kliniec et al., 2023; Snyder et al., 2020). There is also ongoing discussion regarding sunscreen formulation, stability, and real-world effectiveness, particularly under conditions of heat, sweat, and water exposure (Ruszkiewicz et al., 2017; Rosic et al., 2023). These findings highlight the need for clearer, activity-tailored guidance on the combined use of protective clothing and appropriate sunscreen application.

There have been a number of screening studies reporting on KC and MM in sports. Del Boz et al. (2015) have previously reported that 10.3% of a golfing cohort presented with KC and 40% had precancerous skin lesions. More recently, in a cohort of Australian golfers, Stenner et al. (2023) reported that golfers were 2.4 times more likely to receive a skin cancer diagnosis in their lifetime compared to the general population. However, there is a paucity of studies that have included skin examinations with clinicians supported histopathology to confirm the diagnosis as part of the methodology. Dozier et al. (1997) first reported on skin cancer identified in a cohort of Texas surfers, with 16% diagnosed with BCCs and 41% identified with precancerous lesions. No SCC or MM were reported in this surfing cohort (Dozier et al., 1997).

Skin cancers have also been reported in non-aquatic-based cohorts, with Ambros-Rudolph et al. (2006) who identified lesions suggestive of KC alongside increased risk of MM in marathon runners compared to a control group. There are other at-risk occupations (such as working outdoors) for skin cancer that align with the exposure of outdoor enthusiasts. Lichte et al. (2010) reported mountain guides had a higher prevalence of precancerous lesions, KC and MM compared to a control group. Zink et al. (2018) also noted that exposed skin was more likely to have KC in professions primarily focused outdoors.

It has been reported that 63% of MM and virtually all KCs in Australia could be attributed to high background levels of UVR (Olsen et al., 2015). Outdoor physical activity confers substantial health benefits beyond vitamin D synthesis, including reduced risk of several non-skin cancers, improved cardiovascular and metabolic health, and important psychosocial and interpersonal benefits through social engagement and community participation. Outdoor physical activity contributes positively to cardiovascular and psychological well-being and remains a key determinant of vitamin D status. Although vitamin D has been hypothesised to play a protective role in some cancers, prospective evidence does not support a direct protective effect against skin cancer, underscoring the importance of promoting physical activity alongside robust UVR risk mitigation strategies. In support of this, Van der Pols et al. (2013) reported that vitamin D levels were unrelated to skin cancer risk and cautioned that the benefits of high sun exposure do not outweigh the associated dangers. While outdoor recreational activity confers important health benefits, its promotion should be contingent upon concurrent implementation of evidence-based photoprotection measures.

There is some evidence to suggest that media campaigns targeted at educating the importance of sun safe practices within Australia are having an impact on younger populations, with the incidence of skin cancer decreasing in high-risk ancestries (Whiteman et al., 2024; Cust, Scolyer Ao & Long Ao, 2024). Whiteman et al. (2024) reported that the lower incidence of MM in younger populations may be partly explained by increased computer screen time and reduced time spent in outdoor activities. In contrast, the elevated skin cancer rates and the outdoor nature of both aquatic and non-aquatic enthusiasts highlight the vulnerability of these groups. Early detection and treatment are important components of skin cancer control, particularly with regard to reducing morbidity and mortality (Iannacone & Green, 2014). To date, our study is the largest screening study completed in participants who participate in outdoor sports and recreation in an outdoor-focused environment, particularly the aquatic population. This study included highly experienced specialist clinicians with over 10 years’ experience detecting skin cancer, confirmed by histopathology diagnosis.

### Study strengths and limitations

This study represents the largest skin cancer screening of outdoor sport and recreation participants to date in Australia, with over 1,400 participants and robust clinical assessment supported by histopathology, the gold standard for diagnosis. The inclusion of highly experienced clinicians further strengthens diagnostic validity. However, several limitations must be acknowledged. First, classification into aquatic or non-aquatic groups may oversimplify lifetime sun exposure; participants identifying as primarily aquatic may nonetheless accrue greater incidental exposure from land-based activities. For example, a participant may indicate their primary activity spent outdoors was surfing, yet incidental lifetime outdoor exposure may be eclipsed by land-based activities, *i.e.,* walking, cycling, gardening, *etc*.

Second, peak UVR periods were defined as 10 am to 3 pm; however, this window varies geographically and seasonally (*e.g.*, extended exposure in northern Australia compared with southern regions). For example, Darwin in Northern Australia has a UV index above 3 between 8 am and 4 pm year-round, while Hobart in the southernmost point in Australia may struggle to eclipse a UV index of 3 during the winter. Third, participants’ reported sun-protection behaviours may not accurately reflect long-term habits across the lifespan. Furthermore, sun-smart behaviours may have varied over a person’s lifespan, and the clothing and sunscreen application reported in this study may not have accurately reflected the behaviours of some participants during their younger adulthood. Fourth, the majority of the participants in this study had a Fitzpatrick skin type of I-III, resulting in an increased skin cancer risk (Rosic et al., 2023).

Though not a limitation of this study design, there was a bias towards individuals with fairer skin. Finally, we cannot exclude the potential of selection bias as participants in this study attended a clinic focused on detecting skin cancer and melanoma, so by nature, the number of cancerous lesions may be high compared with national numbers. Ethnicity or race was not collected as part of the survey and therefore could not be examined in relation to KC or MM risk. The study population was predominantly of European ancestry, consistent with the demographic profile of patients attending specialist skin cancer clinics in Australia. While this limits exploration of ethnicity-specific risk, it reflects the population at highest risk of MM within the Australian context.

## Conclusions

Aquatic participants exhibited nearly twice the prevalence of MM compared with non-aquatic counterparts, despite reporting significantly lower cumulative sun exposure patterns. This paradox underscores the association between MM prevalence and sun exposure during peak UVR periods, as well as suboptimal adoption of photoprotective strategies. These findings highlight the need for targeted, evidence-based interventions that extend beyond generic sun-safety messaging to include temporal modification of outdoor activity, consistent application of effective photoprotection, and structured surveillance programs. Clinicians should consider outdoor enthusiasts engaged in water-based activities as a higher-risk group for MM, warranting targeted prevention, early detection, and ongoing dermatological follow-up. Outdoor enthusiasts should be recognised as a cohort with a particularly high risk for MM, which should be reflected in national screening policy, and preventative actions should be advised by clinicians and at the local sporting level.

##  Supplemental Information

10.7717/peerj.21278/supp-1 Supplemental Information 1 Participant demographics, preventative measures, skin cancers diagnosed and locations of skin cancers

10.7717/peerj.21278/supp-2 Supplemental Information 2 Categorical variable coding for the published data

10.7717/peerj.21278/supp-3 Supplemental Information 3 STROBE checklist

## Additional Information and Declarations

### Data Availability

The following information was supplied regarding data availability: The data is available in the Supplemental Files.
