# Supplementary material for: Skin cancer prevalence among outdoor activity participants from Queensland, Australia: aquatic versus land-based sun exposure
Source: PeerJ. 2026 Jun 4;14:e21278. doi: 10.7717/peerj.21278 (PMC13242740; doi:10.7717/peerj.21278)
Supplement: Supplemental Information 2 [file peerj-14-21278-s002.docx]

**SKN CANCER AND MELANOMA POINT-PREVALENCE BETWEEN AQUATIC AND NON-AQUATIC ENTHUSIASTS - Supplementary File 3**

**Coding for Published data**

Column B Referral

1 – Self referred

2 – Referred by GP

Column D Activity_Location

1 – Aquatic

2 – Non-aquatic

Column E Gender

1 – Male

2 – Female

Column I Main_Activity_during_peak_UV_yes_or_no

1 – Yes

2 – No

Column K Wear_a_Hat

1 – Yes

2 – No

Column L Wear_a_Rashie

1 – Yes

2 – No

Column M Uses_Sunscreen_OR_Zinc

1 – Yes

2 – No

Column N How_long_ago_last_skin_check

1 – Never

2 – < 6 months

3 – Between 6 months and 1 year

4 – Between 1 and 2 years

5 – Between 2 and 3 years

6 – Between 3 and 4 years

7 – Between 4 and 5 years

8 - > 5 years

Column O FITZPATRICK_Skin_type

1 – Type I

2 – Type II

3 – Type III

4 – Type IV

5 – Type V

6 – Type VI

Column P family_history_SCaMM

1 – Yes

2 – No

Column R Any_skin_lesions_of_concern

1 – Yes

2 – No

Column S Do_you_have_a_history_skin_cancer

1 – Yes

2 – No

Column T Top_of_head_skin_cancer_yesno

1 – Yes

2 – No

Column AJ Face_skin_Ca_yesno

1 – Yes

2 – No

Column AZ lip_skin_Ca

1 – Yes

2 – No

Column BP nose_skin_Ca

1 – Yes

2 – No

Column CF ear_skin_Ca

1 – Yes

2 – No

Column CV neck_skin_Ca

1 – Yes

2 – No

Column DL Abdominal_skin_Ca

1 – Yes

2 – No

Column EB Shoulder_skin_Ca

1 – Yes

2 – No

Column ER Chest_skin_Ca

1 – Yes

2 – No

Column FH Arm_skin_Ca

1 – Yes

2 – No

Column FX Back_skin_Ca

1 – Yes

2 – No

Column GN Hand_skin_cancer

1 – Yes

2 – No

Column HD Up_leg_skin_cancer

1 – Yes

2 – No

Column HT Lower_leg_skin_cancer

1 – Yes

2 – No

Column IJ foot_skin_cancer

1 – Yes

2 – No

Column IZ Any_AK_during_screening

1 – Yes

2 – No

Column JA Any_BCC_Anywhere

1 – Yes

2 – No

Column JC Any_SCC_Insitu_anywhere

1 – Yes

2 – No

Column JE Any_SCC_anywhere

1 – Yes

2 – No

Column JG Combined_SCC_anywhere

1 – Yes

2 – No

Column JI Combined_KC_anywhere

1 – Yes

2 – No

Column JK Any_Melanoma_Anywhere

1 – Yes

2 – No

Column JM Any_Other_Detected

1 – Yes

2 – No
